# Supplementary material for: Topical Cyclosporine in Oral Lichen Planus—A Series of 21 Open-Label, Biphasic, Single-Patient Observations
Source: J Clin Med. 2021 Nov 22;10(22):5454. doi: 10.3390/jcm10225454 (PMC8622196; doi:10.3390/jcm10225454)
Supplement: Supplementary file 1 [file jcm-10-05454-s001.zip › jcm-1449101-supplementary.pdf]

DLQI (Dermatology Life Quality Index) **questionnaire**

DERMATOLOGY LIFE QUALITY INDEX

Hospital No:	Date:		DLQI
Name:		Score:	☐
Address:	Diagnosis:		

The aim of this questionnaire is to measure how much your skin problem has affected your life over the last week. Please tick ⇛ one box for each question.

No.	Question	Answer	Box	
**1.**	Over the last week, how **itchy**, **sore**, **painful** or **stinging** has your skin been?	Very much	❐	
		A lot	❐	
		A little	❐	
		Not at all	❐	
**2.**	Over the last week, how **embarrassed** or **self conscious** have you been because of your skin?	Very much	❐	
		A lot	❐	
		A little	❐	
		Not at all	❐	
**3.**	Over the last week, how much has your skin interfered with you going **shopping** or looking after your **home** or **garden**?	Very much	❐	
		A lot	❐	
		A little	❐	
		Not at all	❐	Not relevant ❐
**4.**	Over the last week, how much has your skin influenced the **clothes** you wear?	Very much	❐	
		A lot	❐	
		A little	❐	
		Not at all	❐	Not relevant ❐
**5.**	Over the last week, how much has your skin affected any **social** or **leisure** activities?	Very much	❐	
		A lot	❐	
		A little	❐	
		Not at all	❐	Not relevant ❐
**6.**	Over the last week, how much has your skin made it difficult for you to do any **sport**?	Very much	❐	
		A lot	❐	
		A little	❐	
		Not at all	❐	Not relevant ❐
**7.**	Over the last week, has your skin prevented you from **working** or **studying**?	Yes	❐	
		No	❐	Not relevant ❐
	If "No", over the last week how much has your skin been a problem at **work** or **studying**?	A lot	❐	
		A little	❐	
		Not at all	❐	
**8.**	Over the last week, how much has your skin created problems with your **partner** or any of your **close friends** or **relatives**?	Very much	❐	
		A lot	❐	
		A little	❐	
		Not at all	❐	Not relevant ❐
**9.**	Over the last week, how much has your skin caused any **sexual difficulties**?	Very much	❐	
		A lot	❐	
		A little	❐	
		Not at all	❐	Not relevant ❐
**10.**	Over the last week, how much of a problem has the **treatment** for your skin been, for example by making your home messy, or by taking up time?	Very much	❐	
		A lot	❐	
		A little	❐	
		Not at all	❐	Not relevant ❐

Please check you have answered EVERY question. Thank you.

©AY Finlay, GK Khan, April 1992 www.dermatology.org.uk, this must not be copied without the permission of the authors.

Table S1. Age range, clinical picture and previous therapies of individual patients at baseline.

No.	Age (years)	Sex	Subtype OLP	cutaneous LP	genital LP	TCS/SCS
1	52	f	erosive	+	-	TCS
2	66	f	erosive	-	+	TCS
3	58	f	erosive	-	-	TCS
4	51	f	atrophic	-	+	TCS
5	56	f	erosive	+	-	TCS
6	58	m	erosive	-	-	TCS
7	73	m	reticular	-	-	TCS
8	69	f	erosive	-	-	TCS
9	40	f	erosive	-	-	SCS, TCS
10	58	m	erosive	-	-	TCS
11	75	f	erosive	-	-	TCS
12	59	f	atrophic	-	-	TCS
13	58	f	erosive	-	-	TCS
14	50	f	erosive	-	-	TCS
15	41	f	erosive	-	-	TCS
16	45	m	reticular	-	+	TCS
17	79	f	erosive	-	-	TCS
18	66	f	erosive	-	-	TCS
19	52	f	erosive	-	-	TCS
20	60	m	atrophic	-	-	TCS
21	78	f	reticular	-	-	TCS

Sex, age, clinical picture and previous therapies of individual patients at baseline. + yes, - no; No.: patient´s number; LP: lichen planus; TCS: topical corticosteroid, SCS: systemic corticosteroid.

Table S2. Wilcoxon rank-sum test to analyse changes between responders and non-responders from T1 (withdrawal of CSA) to T2 (after four weeks without CSA) regarding VAS-, PGA- and DLQI-scores.

Variable	Overall (*n* = 21)<br>No. (%)<br>median (Q1–Q3)	Responder (*n* = 16)<br>No. (%)<br>median (Q1–Q3)	Non-responder (*n* = 5)<br>No. (%)<br>median (Q1–Q3)	*p*-value
VAS Difference T1/T2	1.5 (0–2.5)	1.5 (0–2.8)	1 (0–2.5)	0.90
PGA Difference T1/T2	0.5 (0–1)	0 (0–1)	1 (0.5–1)	0.38
DLQI Difference T1/T2*	0.5 (-0.75–1)	0 (-0.5–1)	1 (-0.5–1.5)	0.75

Wilcoxon rank-sum test to analyse changes between responders and non-responders from T1 (withdrawal of CSA) to T2 (after four weeks without CSA) regarding VAS-, PGA- and DLQI-scores. No significant differences between the two groups were found. * Evaluation of differences in DLQI-scores between T1 and T2 was based on 14 patients.

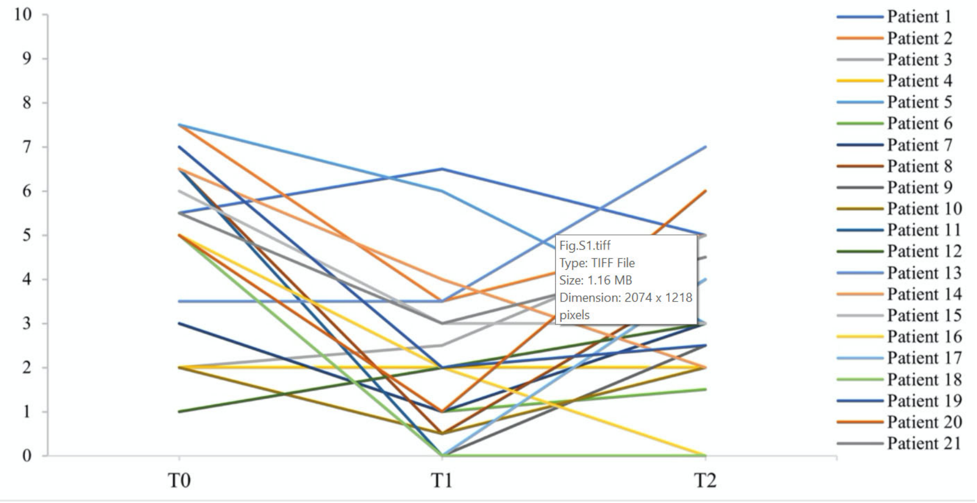

Figure S1. Line graphs showing courses of VAS-scores for each individual patient.
